# Expert views on screening for tuberculosis infection in patients commencing treatment with a biologic agent

**DOI:** 10.36416/1806-3756/e20240082

**Published:** 2024-09-16

**Authors:** Adiba Sultana, Giovanni Battista Migliori, Lia D’Ambrosio, José-María García-García, Denise Rossato Silva, Luis Adrian Rendon, Luigi R Codecasa, Francois-Xavier Blanc, Simon Tiberi, Catherine W M Ong, Courtney Heffernan, Giovanni Sotgiu, Rosella Centis, Claudia Caroline Dobler, The Global Tuberculosis Network

**Affiliations:** 1. University of New South Wales, Sydney, Australia.; 2. The George Institute for Global Health, Sydney, Australia.; 3. Istituti Clinici Scientifici Maugeri - IRCCS - Tradate, Italy.; 4. Public Health Consulting Group, Lugano, Switzerland.; 5. Tuberculosis Research Programme - PII-TB - Spanish Society of Pulmonology and Thoracic Surgery - SEPAR - Barcelona, Spain.; 6. Faculdade de Medicina, Universidade Federal do Rio Grande do Sul - UFRGS - Porto Alegre (RS) Brasil.; 7. Regional TB Reference Centre, Villa Marelli Inst, Niguarda Hosp, Milan, Italy.; 8. Nantes Université, CHU Nantes, Service de Pneumologie, l’institut du thorax, Nantes, France.; 9. Blizard Institute, Barts and The London School of Medicine and Dentistry, Queen Mary University of London, London, United Kingdom.; 10. Infectious Diseases Translational Research Programme, Department of Medicine, Yong Loo Lin School of Medicine, National University of Singapore, Singapore.; 11. Division of Infectious Diseases, Department of Medicine, National University Hospital, Singapore.; 12. Institute for Health Innovation and Technology (iHealthtech), National University of Singapore, Singapore.; 13. University of Alberta, College of Health Sciences, Faculty of Medicine, Edmonton (AB) Canada.; 14. Clinical Epidemiology and Medical Statistics Unit, Department of Medicine, Surgery and Pharmacy, University of Sassari, Italy.

**Keywords:** Latent tuberculosis, Biological products, Immunosuppression therapy, Recurrence, Mass screening

## Abstract

**Objective:**

Many biologic agents cause some degree of immunosuppression, which can increase the risk of reactivation of tuberculosis infection (TBI). This risk is variable between individual biologics. We aimed to assess current (and recommended) clinical practice of TBI screening and treatment among patients initiating treatment with biologic agents.

**Methods:**

An online questionnaire was distributed via email to members of the Global Tuberculosis Network and associated professional organisations to seek insights into the screening for and treatment of TBI in patients treated with biologics.

**Results:**

A total of 163 respondents in 27 countries answered at least one question. For all biologics described in the questionnaire, respondents advised increasing screening relative to current practice. Observed and supported TBI screening rates in patients treated with TNF-a inhibitors were high, especially for older TNF-a inhibitors. Most participants supported TBI screening in patients treated with B- or T-cell inhibitors but not in those treated with interleukin inhibitors. Guideline awareness was higher for TNF-a inhibitors than for other biologic classes (79% vs. 34%).

**Conclusions:**

Although respondents stated that TBI screening rates are lower than what they consider ideal, there was a tendency to recommend TBI screening in patients treated with biologics not known to be associated with an increased risk of TBI. As a result, there is a potential risk of over-screening and over-treatment of TBI, potentially causing harm, in patients treated with biologics other than TNF-a inhibitors. There is a need to research the risk of TBI associated with biologics and for guidelines to address the spectrum of TBI risk across all types of biologics.

## INTRODUCTION

Tuberculosis infection (TBI) results from airborne spread of the bacterium *Mycobacterium tuberculosis* from a contagious patient to a susceptible individual. Once infected, the normal host immune response is to confine the TB bacteria within the lung. In most patients, this infection remains confined-a condition previously described as latent TB infection and latterly as TBI. For approximately 5-15% of people with TBI (lifetime risk), the bacteria can escape confinement resulting in active tuberculosis. Although there is not always an identifiable trigger for reactivation, it is significantly more common in immunocompromised individuals.^1^

Biologics, also known as biopharmaceuticals, are drugs containing components from living organisms and typically work by suppressing aspects of the immune system, thus increasing the risk of tuberculosis reactivation. As shown in Table 1, there are four main classes of biologic agents^2^ ^-^ ^14^: TNF-a inhibitors, interleukin inhibitors, T-cell inhibitors and B-cell inhibitors. The risk of tuberculosis reactivation associated with different medications differs, even within a given class.

**Table 1:** Risk of tuberculosis infection associated with different biologic drugs.

Class	Name	TBI reactivation risk in comparison with the general population	Risk in comparison with other drugs or population subgroups
		RR (95% CI)	RR (95% CI)
TNF-a inhibitors	All classes	17.1 (13.9-21.0)^3^	4.0 (2.4-6.9), compared with patients with RA taking non-biologic disease modifying agents^3^
	Infliximab	18.6 (13.4-25.8), based on the SIR^4^	2.8 (2.1-3.7), compared with etanercept^3^
	Adalimumab	29.3 (20.3-42.4), based on the SIR^4^	3.9 (2.3-6.5), compared with etanercept^3^
	Etanercept	1.8 (0.7-4.3), based on the SIR^4^	
	Golimumab	Insufficient data^5^	Biologic class
	Certolizumab pegol	No comparable data	Similar reactivation risk compared with the other TNF-a inhibitor drugs^6^
T-cell inhibitors	Abatacept	No comparable data^7^; No significant increase^5^ ^,^ ^8^	Lower compared with TNF-a inhibitors^8^
B-cell inhibitors	Rituximab	No increase; no known cases^5^ ^,^ ^9^ ^,^ ^10^	Lower compared with TNF-a inhibitors^8^
Interleukin inhibitors			
IL-6Ra inhibitor	Sarilumab	Insufficient data^8^	
IL-6 inhibitor	Tocilizumab	Low^5^ ^,^ ^11^	
IL-5 inhibitor	Mepolizumab	No increase^12^ ^,^ ^13^	
IL-5Ra inhibitor	Benralizumab	No increase^12^	
IL-12/IL-23 inhibitor	Ustekinumab	No increase^8^ ^,^ ^11^ ^,^ ^14^	
IL-17 inhibitor	Secukinumab	Slightly elevated^8^	

TBI: tuberculosis infection; RA: rheumatoid arthritis; SIR: standardised incidence ratio.

The increased risk of active tuberculosis associated with TNF-a inhibitors has been well documented.^14^ There is, however, a paucity of meaningful data about the risk of active tuberculosis associated with biologics other than TNF-a inhibitors. Studies comparing the risk of active tuberculosis in patients treated with a specific biologic with that determined for the general population (in the same setting) would allow the relative risk of active tuberculosis associated with biologic use to be estimated.^15^ ^,^ ^16^ What evidence is currently available, however, suggests that the risk of tuberculosis reactivation by most non-TNF-a inhibitors is not likely significant (See Table 1).

The risk of TBI reactivation associated with B-cell inhibitor use is thought to be low because the tuberculosis immune response is largely T-cell dominated.^5^ ^,^ ^9^ Rituximab is a widely used and researched B-cell inhibitor, with all available evidence indicating a zero to negligibly elevated risk of TBI reactivation.^5^ ^,^ ^9^ ^,^ ^11^ As a result, a Rituximab Consensus Expert Committee in rheumatology recently determined TBI screening to be unnecessary prior to its initiation.^5^ ^,^ ^17^

Although the tuberculosis immune response is largely T-cell driven, use of the T-cell inhibitor abatacept has not been associated with an increased relative risk of TBI reactivation.^5^ ^,^ ^8^ There is scant information available regarding the risk of TBI reactivations associated with T-cell inhibitors other than abatacept.

Where data are available (Table 1), they suggest that the risk of TBI reactivation associated with the use of interleukin inhibitors is either minimally elevated or non-existent.^5^ ^,^ ^8^ ^,^ ^11^ ^-^ ^14^ ^,^ ^18^ ^-^ ^20^

Despite reported differences in the risk of TBI reactivation associated with different biologics, it is unclear whether this variability is taken into consideration by physicians determining the need for TBI screening and treatment. There are currently only a few guidelines on TBI screening in patients treated with biologics other than TNF-a inhibitors, with most being generally based on poor quality of evidence (see supplementary material: Table S1). Anecdotal evidence suggests that TBI screening practices fluctuate significantly across different health care facilities and sometimes even between providers within the same facility.

The primary objective of this paper is to describe current clinical practice regarding TBI screening and treatment of patients initiating treatment with biologic agents, on the basis of the results obtained with a survey distributed to members of the Global TB Network. A secondary objective is to identify areas of significant variation in screening practices, which should be addressed with updated clinical practice guidelines.

## METHODS

### Ethical considerations

Ethics approval for the survey was obtained from the South Western Sydney Local Health District Human Research Ethics Committee (Reference no. 2021/ETH12298).

### Survey design

The study survey was designed with Qualtrics software (https://www.qualtrics.com/free-account/). Questions were organised into several sections exploring different aspects of TBI screening and treatment. We sought to achieve an understanding of current and ideal practice, as considered by experts in the topic of tuberculosis. Demographic data, including age, gender, country of birth and country of practice, were also collected.

The biologic agents listed in the survey were selected to represent indications for various diseases and different lengths of time available on the market. All biologics listed are approved for clinical use by the European Medicine Agency and the U.S. Food and Drug Administration.

Five participants filled out a pilot questionnaire. On the basis of the feedback received at this step, some minor changes were made. Responses to the pilot surveys were included in the final data analysis.

### Survey distribution

An email that included a link to the survey, with a participant information sheet attached, was sent to members of the Global TB Network. The Global TB Network is an international group of healthcare practitioners involved in tuberculosis care, including clinicians, epidemiologists and researchers.^21^ Members of the Global TB Network also distributed our survey within their own networks, including the Brazilian Thoracic Association, the Spanish Society of Pulmonology and Thoracic Surgery (SEPAR), the Mexican Pulmonary and Thoracic Surgery Society, the French Society of Respiratory Diseases, the Canadian TB Elimination Network and the Australian TB Forum. Participants were informed that their participation in this project was entirely voluntary, and that submission of the survey would imply their informed consent.

### Data collection and analysis

The survey was open between 2 May of 2022 and 21 July of 2022. Responses were collected in Qualtrics and exported to Microsoft Excel to facilitate descriptive analyses.

Proportions of responses were calculated for each question, meaning that the denominator could vary between questions. An overall response rate could not be calculated because of the network sampling technique that was employed to distribute surveys. However, rates for fully vs. partially complete surveys were calculated and are described below.

Participants were grouped based on the tuberculosis incidence in their country of practice. Low-incidence settings were distinguished from intermediate-to-high-incidence settings using a cut-off of an annual tuberculosis incidence of 40 per 100,000 population. National tuberculosis incidence rates for 2022 were obtained from the World Health Organization (WHO) website.^22^

## RESULTS

**Figure 1:**
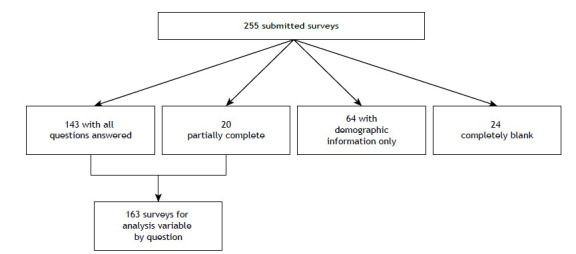
Surveys considered for analysis.

A total of 255 surveys were returned to us from participants in a total of 27 countries (Figure 1). Of those, 163 were categorized as complete or partially complete. A survey was considered partially complete if there was a response to at least one question after the demographics section but not all questions were answered.

Of the 163 complete or partially complete surveys, 100 (61.3%) were from European countries (see supplementary material: Figure S1). Grouping country of practice by tuberculosis incidence, 16 countries were included in the low-incidence category, which comprised 114 participants. The remaining 10 countries were categorised as intermediate-to-high-incidence countries and comprised 48 participants. One survey respondent did not provide their country of practice.

Current reported screening practices for TBI as well as screening practices judged as optimal by participants varied among the biologics surveyed (Table 2). There were high rates of observed screening for the TNF-a inhibitor class, especially for the older drugs infliximab (93%), adalimumab (89%) and etanercept (88%). The perceived screening rates for certolizumab pegol and golimumab were lower (67% and 68%, respectively). For TBI screening in patients treated with TNF-a inhibitors, participant recommendations for using an IFN-gamma release assay (IGRA) or tuberculin skin test (TST) ranged from 88% and 97%, and recommendations for using chest X-ray (CXR) in the screening process ranged from 55% to 61%.

**Table 2:** Currently observed practices regarding screening for tuberculosis infection and screening practices suggested, for different biologics.

Class/biologic	Participant responses						
	Currently screens for TBI	Supports the use of IGRA and TST for TBI screening	Supports the use of CXR for TBI screening	Thinks that TBI screening is warranted	Thinks there is insufficient evidence for TBI screening	Does not know whether TBI screening is indicated	Thinks any form of TBI screening (IGRA/TST/CXR) is warranted
	% (n/N)	% (n/N)	% (n/N)	% (n/N)	% (n/N)	% (n/N)	% (n/N)
TNF-a inhibitors							
Infliximab	93.2 (151/162)	96.2 (151/157)	61.1 (96/157)	0	0	3.2 (5/157)	96.8 (152/157)
Adalimumab	89.4 (143/160)	96.7 (145/150)	60.0 (90/150)	0	0	2.7 (4/150)	98.0 (147/150)
Etanercept	87.6 (141/161)	96.6 (143/148)	60.8 (90/148)	0	0	2.0 (3/148)	98.0 (145/148)
Certolizumab pegol	66.4 (103/155)	88.2 (127/144)	54.9 (79/144)	0	0.7 (1/144)	11.1 (16/144)	89.6 (129/144)
Golimumab	68.2 (107/157)	89.5 (128/143)	55.2 (79/143)	0	0.7 (1/143)	9.8 (14/143)	90.9 (130/143)
T-cell inhibitor							
Abatacept	59.6 (90/151)	70.7 (99/140)	43.6 (61/140)	2.8 (4/140)	5.0 (7/140)	20.0 (28/140)	72.8 (102/140)
B-cell inhibitor							
Rituximab	59.5 (94/158)	61.3 (87/142)	39.4 (56/142)	14.8 (21/142)	11.3 (16/142)	10.6 (15/142)	64.1 (91/142)
Interleukin inhibitors							
Sarilumab (IL-6)	38.0 (57/150)	52.5 (74/141)	31.2 (44/141)	7.1 (10/141)	17.0 (24/141)	23.4 (33/141)	53.9 (76/141)
Tocilizumab (IL-6)	47.4 (72/152)	58.2 (82/141)	36.2 (51/141)	11.3 (16/141)	17.0 (24/141)	13.5 (19/141)	60.3 (85/141)
Mepolizumab (IL-5)	17.2 (26/151)	29.0 (40/138)	19.6 (27/138)	31.2 (43/138)	18.1 (25/138)	19.6 (27/138)	33.3 (46/138)
Benralizumab (IL-5)	16.0 (24/150)	27.5 (38/138)	18.1 (25/138)	31.2 (43/138)	18.1 (25/138)	20.3 (28/138)	31.9 (44/138)
Ustekinumab (IL-12/IL-23)	47.0 (72/153)	47.8 (67/140)	32.9 (46/140)	10.7 (15/140)	15.7 (22/140)	25.7 (36/140)	50.0 (70/140)
Secukinumab (IL-17)	36.6 (56/153)	43.1 (59/137)	28.1 (43/153)	9.5 (13/137)	18.2 (25/137)	29.2 (40/137)	46.0 (63/137)

TBI: tuberculosis infection; IGRA: interferon-gamma release assay; TST: tuberculin skin test; and CXR: chest X-ray.

Most respondents reported and supported TBI screening in patients treated with B- or T-cell inhibitors but not in patients treated with interleukin inhibitors except those treated with sarilumab or tocilizumab, for which more than 50% recommended TBI screening despite a minority believing that this is current practice at their health care facility. The rates of observed and suggested TBI screening were lowest for the severe asthma drugs benralizumab and mepolizumab (16% and 17%, respectively). Those two drugs also had the highest proportions of respondents advising against screening (31% for both).

For all biologics, overall, the number of participants who considered a CXR to be warranted in the TBI screening process was lower than that of those who recommended the use of an IGRA or TST (Table 2). For all of the biologics listed, the respondents supported more screening than what they currently observed at their respective health care facilities.

Of the 159 participants who answered the relevant question, 126 (79.2%) were aware of at least one clinical guideline for TBI screening in patients initiating treatment with a TNF-a inhibitor, compared with 51 (34.4%) of the 148 participants who answered the question related to guidelines for patients initiating treatment with a non-TNF-a inhibitor.

Of the 84 participants who could name at least one guideline relating to TBI screening prior to TNF-a inhibitor use, 20 named the WHO guidelines and 9 named the SEPAR guidelines. Other named guidelines included those issued by the Centers for Disease Control and Prevention (n = 4), the European Society of Clinical Microbiology and Infectious Disease (ESCMID; n = 3), the British Thoracic Society (n = 3) and the American Thoracic Society (n = 2). Three participants named systematic reviews: two reviews of international guidelines^23^ ^,^ ^24^; and one that was specific for non-TNF-a biologics.^25^ Twenty-four participants (19%) were aware of local hospital guidelines. Multiple participants named national guidelines from their country of practice, including Brazil (n = 8) and France (n = 8).

Of the 25 participants who named at least one guideline for non-TNF-a inhibitors, 5 were aware of the SEPAR guidelines and 2 were aware of the ESCMID guidelines. Ten participants named local or national guidelines from their country of practice. Other named guidelines included those issued by the WHO (n = 3) and the Centers for Disease Control and Prevention (n = 1), as well as those issued by Alimentary Pharmacology and Therapeutics (n = 1). Two participants named vague guidelines, such as “rheumatological guidelines” and “clinical recommendation 2021”, and one participant named the systematic review specific for non-TNF-a biologics.^25^

**Figure 2:**
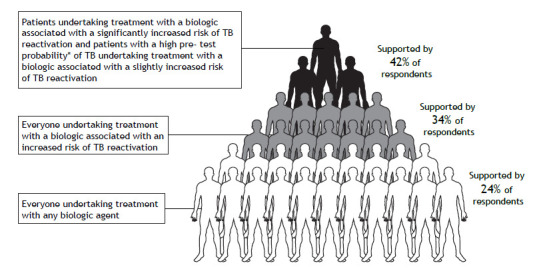
Proportional distribution of participant responses regarding which patients initiating treatment with which biologics should be screened for infection with tuberculosis (TB). *An increased pre-test probability would, for example, be based on a history of close TB contact, birth in a high TB burden country, and previous treatment for active tuberculosis. NOTE: This figure was created by using Servier Medical Art templates, which are licensed under a Creative Commons Attribution 3.0 Unported License; https://smart.servier.com.

Most respondents were selective when choosing patients for TBI screening (Figure 2). However, a considerable proportion (23.8%) suggested that TBI screening should be conducted in all patients treated with biologics irrespective of the TBI reactivation risk associated with the biologic in question. The number of participants who recommended any form of TBI screening for any of the biologics listed was higher than was that of those who recommended no screening (see supplementary material: Table S5 ).

There were two preferred screening algorithms, with 36.0% of the participants being in favour of IGRA alone and 33.3% favouring IGRA and TST sequentially (in any order) if the first test result is negative. Only a few participants (4%) supported the use of TST alone.

Most (60.1%) of the respondents were in favour of a CXR being performed in all patients undergoing treatment with biologic agents independent of the result of TBI screening or the presence of symptoms (Table 3). A CXR can help to exclude active tuberculosis and detect other diagnoses, such as a neoplasm.

**Table 3:** Survey participant suggestions regarding the role of chest X-ray in screening for tuberculosis infection in patients initiating treatment with a biologic.

Under what condition CXR should be performed	(N = 148)
In all patients, n (%)	89 (60.1)
Only when TBI screening is positive, n (%)	25 (16.9)
Only in patients with symptoms (e.g., cough), n (%)	9 (6.1)
In symptomatic patients or when TBI screening is positive, n (%)	25 (16.9)

CXR: chest X-ray; and TBI: tuberculosis infection.

Of the 146 participants who answered the relevant question, 114 (78.0%) indicated that they would repeat TBI screening upon new exposure to an infectious tuberculosis patient, whereas only 63 (43.2%) indicated that they would repeat screening upon travel to a high tuberculosis incidence country and only 52 (35.6%) indicated that they would perform TBI screening at regular intervals. In the free text response, four survey participants emphasised the need to know the baseline screening result before making any decisions about tests (see supplementary material: Tables S2 and S3).

The most commonly used tuberculosis preventive therapy regimen, reported by 43.6% of the respondents, was 6-9 months of isoniazid (Table 4), whereas 45.7% indicated a preference for rifampin-containing regimens, either alone for 4 months (25.0%) or together with isoniazid for 3 months (20.7%). The least commonly used treatment regimen (cited by 9.3% of the respondents) was 12 doses of rifapentine and isoniazid over 3 months.

**Table 4:** Tuberculosis preventive therapy preferences amongst respondents, by tuberculosis incidence level in the country of practice.

Tuberculosis incidence	Preferred treatment regimen							
	6-9 months of isoniazid	4 months of rifampin	3 months (12 doses) of rifapentine + isoniazid	3 months of isoniazid + rifampin	Other	Total responses	No response	Grand total
	% (n/N)	% (n/N)	% (n/N)	% (n/N)	% (n/N)	N	n	N
Low	40.0 (40/100)	29.0 (29/100)	4.0 (4/100)	27.0 (27/100)	0	100	14	114
Intermediate to high	53.8 (21/39)	15.4 (6/39)	20.5 (8/39)	5.1 (2/39)	5.1 (2/39)	39	9	48
Unknown*	0	0	0	0	100.0 (1/1)	1	0	1
Total	43.6 (61/140)	25.0 (35/140)	8.6 (12/140)	20.7 (29/140)	2.1 (3/140)	140	23	163

*Country of practice not indicated.

Of the 102 participants in low tuberculosis incidence countries, 71 (69.6%) preferred patients to undergo 1 month of tuberculosis treatment prior to the start of treatment with a biologic agent (see supplementary material: Table S4). However, only 22 (56.4%) of the 39 respondents in intermediate-to-high tuberculosis incidence countries were in favour of this approach, generally recommending a longer duration of tuberculosis treatment before commencing treatment with a biologic compared with those in low incidence countries. Few participants selected the “Other” option, in which some emphasised that the duration of tuberculosis treatment would be variable depending on the urgency of biologic treatment (n = 4) and some suggested immediate or concurrent commencement of treatment with the biologic (n = 3).

Of the 141 participants who answered the relevant question, 114 (80.8%) supported monitoring of liver function test results during tuberculosis treatment. Support was very low for routine repeat CXR (5.7%) and repeat TBI screening (4.2%), whereas 14.9% preferred to conduct no routine monitoring tests.

For the 10 participants who chose the free text option, recommendations included a complete blood count (n = 4), renal function tests (n = 2) and monitoring clinical symptoms or adverse events (n = 2). The 2 remaining participants emphasised the importance of the baseline TBI screening results.

A comparison of the survey responses from each country and their national guidelines showed that there was some deviation between the two. Although the SEPAR guidelines recommend screening for patients treated with any biologic, 70% of the participants working in Spain would not screen patients treated with rituximab (which aligns with other international guidelines). Most (66%) of those participants follow the guidelines to initiate biologics after 1 month of tuberculosis treatment and an even larger proportion (87%) would repeat TBI screening upon exposure to an infectious tuberculosis patient, although only 57% adhered to the guideline recommendation for using a combination of IGRA and TST during screening for TBI. There was more variation in the chosen treatment regiments for TBI, with 38% following the SEPAR recommendation of 6-9 months of isoniazid and 31% suggesting 3 months of the rifampin-isoniazid combination, which the guidelines recommend in exceptional circumstances only (see supplementary material: Table S1). Many (43%) of the participants in Brazil showed a preference for using either TST or IGRA without preference, as opposed only 14% who stated that they adhere to the Brazilian Thoracic Association recommendation for TST only. The Brazilian guidelines were published in 2009, possibly explaining this discrepancy. Specifically, the guidelines recommend periodic TST testing in patients initiating treatment with TNF-a inhibitors (see supplementary material: Table S1). Most (64%) of the participants in Brazil were aligned with their national guidelines in terms of their tuberculosis treatment of choice, employing the recommended 6-9 months of isoniazid, and in terms of the timing of the initiation of biologic treatment, following the recommendation of waiting until after 1 month of tuberculosis treatment. Participants in Australia and the United Kingdom (8 participants from each) generally followed their national guidelines. From Europe, North America, Asia and Oceania, there was only a limited number of completed surveys, most of which were from Europe. That reflects the profile of the membership of the Global TB Network which has a strong European base. 

## DISCUSSION

Management of TBI is a core intervention to achieve tuberculosis elimination, with patients treated with TNF-a inhibitors and other biologics representing a vulnerable group deserving specific attention.^26^

The results of this global survey suggest that tuberculosis specialists believe that there is under-screening of patients treated with different biologics at their respective health care facilities. There was strong support for TBI screening in patients treated with TNF-a inhibitors, as well as a high level of awareness of at least one clinical practice guideline for TBI screening in patients initiating treatment with TNF-a inhibitors. Participant awareness of guidelines regarding TBI screening in patients initiating treatment with non-TNF-a inhibitor biologics was much lower. There was also a high degree of variation in current screening practices for the non-TNF-a inhibitors other than mepolizumab and benralizumab. Those two biologics are commonly used for the treatment of severe asthma and were associated with low rates of screening recommendations by the respondents. Most respondents reported and supported TBI screening in patients treated with B- or T-cell inhibitors but not in patients treated with all interleukin inhibitors (the exceptions being sarilumab and tocilizumab). The most popular screening regimen was for IGRA alone or IGRA and TST used sequentially (in any order) if the first result is negative. There were no substantial differences in tuberculosis screening recommendations between low- and intermediate-to-high-incidence countries.

The increased risk of tuberculosis associated with TNF-a inhibitors is well documented, and it seems that many health care professionals extrapolate that risk to other biologic classes.^5^ ^,^ ^27^ In our study, respondents indicated very high perceived levels of TBI screening for TNF-a inhibitors and supported such screening, with no participants recommending against it. This is consistent with current evidence of the significantly elevated TBI reactivation risk associated with these drugs.^3^ It is noteworthy that, despite evidence suggesting a low or possibly absent risk of TBI reactivation with non-TNF-a inhibitors, the proportion of participants preferring any form of TBI screening was greater than was that of those preferring no screening for patients treated with any of the biologics listed in this survey.

The guidelines and clinical standards currently available^28^ mainly focus on TBI screening in patients treated with TNF-a inhibitors and to a much lesser extent on screening in patients treated with other biologics. Some, such as the SEPAR guidelines,^29^ extrapolate the recommendations for TNF-a inhibitors to other biologics. The ESCMID guidelines explicitly refer to different biologics by name,^13^ ^,^ ^30^ with separate risk assessments for specific classes of biologic agents. Local guidelines from the National Health Service Gloucestershire Hospitals and the Drug and Bulletin Board of Navarre, Spain, also give specific recommendations for individual biologics.^31^ ^,^ ^32^ All guidelines are generally based on weak or insufficient evidence.

The general recommendation for TBI screening in our survey seemed to be relatively undifferentiated for different biologic classes and not necessarily aligned with the low TBI reactivation risk for many non-TNF-a inhibitor biologics. However, these recommendations were often aligned with guideline recommendations. Although evidence suggests that ustekinumab is associated with no increased risk of TBI reactivation, multiple guidelines recommend screening in patients treated with this medication.^11^ ^,^ ^14^ The ESCMID guidelines justify this by stating that there is a biologically plausible increase in TBI reactivation risk.^13^ Though no cases of TBI reactivation have been associated with the use of sarilumab, the ESCMID guidelines recommend screening because it is an IL-6 inhibitor like tocilizumab, which has been associated with a risk, albeit a low risk, of TBI reactivation.^11^ ^,^ ^13^ ^,^ ^20^ This clearly demonstrates the need for high quality studies assessing the risk of TBI reactivation associated with different biologics, to inform the development of guidelines.

The only biologics included in this study for which some guidelines (including those at a local level) recommended against TBI screening were rituximab, mepolizumab and benralizumab. Current evidence suggests that these three drugs, along with ustekinumab, are not associated with an increased risk of TBI reactivation.^5^ ^,^ ^8^ ^,^ ^9^ ^,^ ^11^ ^-^ ^14^ The survey results demonstrate considerable variation in screening practices for rituximab (60% current observed screening) and ustekinumab (47% current observed screening). Almost equal proportions of participants indicated they believe any form of TBI screening is indicated for patients treated with mepolizumab or benralizumab (33% and 32%, respectively, vs. 31% who believed that no screening is indicated for either drug), which indicates that there is clinical uncertainty and variation in practice.

Participants supported more frequent screening than what they currently observed for all of the drugs listed in this study. A universal recommendation for TBI screening in patients treated with any biologic can lead to over-screening, an increased risk of false-positive test results if the pre-test tuberculosis risk is low and subsequent over-treatment. This may unnecessarily expose the patient to the potential adverse effects of TBI treatment, mainly hepatotoxicity.^33^ Therefore, it is important that the risks and benefits of TBI screening and treatment are assessed on an individual basis when dealing with patients treated with biologics other than TNF-a inhibitors.

We found that current practice did not always align with national guidelines regarding screening for TBI in patients about to receive biologics. In some countries, the national guidelines had not been updated recently, which could explain such divergences.

Our study has some limitations. The smaller number of responses from tuberculosis professionals working in countries with a high tuberculosis incidence likely reflects the different approach to tuberculosis control in those countries. In high-incidence countries, treatment of active tuberculosis rather than tuberculosis preventive therapy is the primary emphasis of tuberculosis control programmes. In addition, at the time of the distribution of the survey, the combination treatment of isoniazid and rifapentine was not widely available in some countries, and it is unclear whether this TBI treatment regimen would be the preferred option for many today. That combination was also not listed in most (older) guidelines as a preferred treatment regimen. The descriptive study analysis provides insights into international practices but does not allow us to establish causality (e.g., between observed practice and local/national guidelines). Furthermore, although the participants were internationally recognised tuberculosis experts, their views may not necessarily be representative of the practices and recommendations related to tuberculosis in their country of practice.

This study has demonstrated participant uncertainty about the need for tuberculosis screening in patients treated with biologics other than TNF-a inhibitors, for which there has been little research and there are fewer available guidelines than for TNF-a inhibitors. Where guidelines exist, they are based on weak or insufficient evidence and are often informed by expert opinion. Therefore, there is a need for further studies of the TBI reactivation risk associated with different biologic agents such as T-cell inhibitors and interleukin inhibitors. In addition, this study illustrates the need for evidence-based clinical guidelines to be developed and disseminated amongst clinicians, along with clear recommendations addressing what to do when there is insufficient information. Recommendations for or against TBI screening should be considered for all immunosuppressive drugs not just biologics.

## References

[1] Migliori GB, Ong CWM, Petrone L, D'Ambrosio L.Centis R.Goletti D (2021). The definition of tuberculosis infection based on the spectrum of tuberculosis disease. Breathe (Sheff).

[2] Ritter J, Flower RJ, Henderson G, Loke YK, MacEwan D, Rang HP (2020). Rang & Dale's Pharmacology.

[3] Ai JW, Zhang S, Ruan QL, Yu YQ, Zhang BY, Liu QH (2015). The Risk of Tuberculosis in Patients with Rheumatoid Arthritis Treated with Tumor Necrosis Factor-a Antagonist A Metaanalysis of Both Randomized Controlled Trials and Registry/Cohort Studies. J Rheumatol.

[4] Tubach F, Salmon D, Ravaud P, Allanore Y, Goupille P, Bréban M (2009). Risk of tuberculosis is higher with anti-tumor necrosis factor monoclonal antibody therapy than with soluble tumor necrosis factor receptor therapy The three-year prospective French Research Axed on Tolerance of Biotherapies registry [published correction appears in Arthritis. Rheum.

[5] Cantini F, Niccoli L, Goletti D (2014). Tuberculosis risk in patients treated with non-anti-tumor necrosis factor-a (TNF-a) targeted biologics and recently licensed TNF-a inhibitors data from clinical trials and national registries. J Rheumatol Suppl.

[6] Curtis JR, Mariette X, Gaujoux-Viala C, Blauvelt A, Kvien TK, Sandborn WJ (2019). Long-term safety of certolizumab pegol in rheumatoid arthritis, axial spondyloarthritis, psoriatic arthritis, psoriasis and Crohn's disease a pooled analysis of 11 317 patients across clinical trials. RMD Open.

[7] Simon TA, Dong L, Winthrop KL (2021). Risk of opportunistic infections in patients with rheumatoid arthritis initiating abatacept cumulative clinical trial data. Arthritis Res Ther.

[8] Evangelatos G, Koulouri V, Iliopoulos A, Fragoulis GE (2020). Tuberculosis and targeted synthetic or biologic DMARDs, beyond tumor necrosis factor inhibitors. Ther Adv Musculoskelet Dis.

[9] Alkadi A, Alduaiji N, Alrehaily A (2017). Risk of tuberculosis reactivation with rituximab therapy. Int J Health Sci (Qassim).

[10] Shobha V, Chandrashekara S, Rao V, Desai A, Jois R, Dharmanand BG (2019). Biologics and risk of tuberculosis in autoimmune rheumatic diseases A real-world clinical experience from India. Int J Rheum Dis.

[11] Cantini F, Nannini C, Niccoli L, Petrone L, Ippolito G, Goletti D (2017). Risk of Tuberculosis Reactivation in Patients with Rheumatoid Arthritis, Ankylosing Spondylitis, and Psoriatic Arthritis Receiving Non-Anti-TNF-Targeted Biologics. Mediators Inflamm.

[12] Liu AY (2020). Infectious Implications of Interleukin-1, Interleukin-6, and T Helper Type 2 Inhibition. Infect Dis Clin North Am.

[13] Winthrop KL, Mariette X, Silva JT, Benamu E, Calabrese LH, Dumusc A (2018). ESCMID Study Group for Infections in Compromised Hosts (ESGICH) Consensus Document on the safety of targeted and biological therapies an infectious diseases perspective (Soluble immune effector molecules [II]: agents targeting interleukins, immunoglobulins and complement factors). Clin Microbiol Infect.

[14] Dobler CC (2016). Biologic Agents and Tuberculosis. Microbiol Spectr.

[15] Campbell C, Andersson MI, Ansari MA, Moswela O, Misbah SA, Klenerman P (2021). Risk of Reactivation of Hepatitis B Virus (HBV) and Tuberculosis (TB) and Complications of Hepatitis C Virus (HCV) Following Tocilizumab Therapy A Systematic Review to Inform Risk Assessment in the COVID-19 Era. Front Med (Lausanne).

[16] Kelsey A, Chirch LM, Payette MJ (2018). Tuberculosis and interleukin blocking monoclonal antibodies Is there risk?. Dermatol Online.

[17] Buch MH, Smolen JS, Betteridge N, Breedveld FC, Burmester G, Dörner T (2011). Updated consensus statement on the use of rituximab in patients with rheumatoid arthritis. Ann Rheum Dis.

[18] Romiti R, Valenzuela F, Chouela EN, Xu W, Pangallo B, Moriarty SR (2019). Prevalence and outcome of latent tuberculosis in patients receiving ixekizumab integrated safety analysis from 11 clinical trials of patients with plaque psoriasis. Br J Dermatol.

[19] Fowler E, Ghamrawi RI, Ghiam N, Liao W, Wu JJ (2020). Risk of tuberculosis reactivation during interleukin-17 inhibitor therapy for psoriasis a systematic review. J Eur Acad Dermatol Venereol.

[20] Lee EB (2018). A review of sarilumab for the treatment of rheumatoid arthritis. Immunotherapy.

[21] Silva DR, Rendon A, Alffenaar JW, Chakaya JM, Sotgiu G, Esposito S (2018). Global TB Network working together to eliminate tuberculosis. J Bras Pneumol.

[22] World Health Organization (2022). Global Tuberculosis Report 2022.

[23] Cantini F, Nannini C, Niccoli L, Iannone F, Delogu G, Garlaschi G (2015). Guidance for the management of patients with latent tuberculosis infection requiring biologic therapy in rheumatology and dermatology clinical practice. Autoimmun Rev.

[24] Federatie Medisch Specialisten [homepage on the Internet] Risico-inventarisatie op latente tbc-infectie.

[25] Diel R, Schaberg T, Nienhaus A, Otto-Knapp R, Kneitz C, Krause A (2021). Joint Statement (DZK, DGRh, DDG) on the Tuberculosis Risk with Treatment Using Novel Non-TNF-Alpha Biologicals. Pneumologie.

[26] Migliori GB, Dowdy D, Denholm JT, D'Ambrosio L, Centis R (2023). The path to tuberculosis elimination a renewed vision. Eur Respir J.

[27] Harris J, Keane J (2010). How tumour necrosis factor blockers interfere with tuberculosis immunity. Clin Exp Immunol.

[28] Migliori GB, Wu SJ, Matteelli A, Zenner D, Goletti D, Ahmedov S (2022). Clinical standards for the diagnosis, treatment and prevention of TB infection. Int J Tuberc Lung Dis.

[29] Mir Viladrich I, Daudén Tello E, Solano-López G, López Longo FJ, Taxonera Samso C, Sánchez Martínez P (2016). Consensus Document on Prevention and Treatment of Tuberculosis in Patients for Biological Treatment. Arch Bronconeumol.

[30] Mikulska M, Lanini S, Gudiol C, Drgona L, Ippolito G, Fernández-Ruiz M (2018). ESCMID Study Group for Infections in Compromised Hosts (ESGICH) Consensus Document on the safety of targeted and biological therapies an infectious diseases perspective (Agents targeting lymphoid cells surface antigens [I]: CD19, CD20 and CD52). Clin Microbiol Infect.

[31] Rodriguez I (2020). I, Zamarbide OG Risk of Infection Associated with Biological Agents Used for Autoimmune Inflammatory Diseases. Drug Therapeut Bull Navarre.

[32] White A, Terry L (2023). Guideline for Tuberculosis screening for Biologic and Immunomodulatory drugs for inflammatory conditions.

[33] Saukkonen JJ, Cohn DL, Jasmer RM, Schenker S, Jereb JA, Nolan CM (2006). An official ATS statement hepatotoxicity of antituberculosis therapy. Am J Respir Crit Care Med.

